# National audit of cognitive assessment in people with pwMND A national audit of cognitive assessment in people with motor neurone disease (pwMND) in Scotland

**DOI:** 10.1080/21678421.2020.1752249

**Published:** 2020-04-20

**Authors:** Maria Stavrou, Judith Newton, Gill Stott, Shuna Colville, Siddharthan Chandran, Sharon Abrahams, Suvankar Pal, Richard Davenport

**Affiliations:** 1 Department of Clinical Neurosciences, NHS Lothian, Edinburgh, UK,; 2 Euan Macdonald Centre of MND Research, Edinburgh, UK,; 3 Anne Rowling Regenerative Neurology Clinic, Edinburgh, UK,; 4 Centre of Clinical Brain Sciences, University of Edinburgh, Edinburgh, UK, and; 5 Clinical Audit Research and Evaluation for Motor Neurone Disease, Scotland, UK

**Keywords:** Motor neurone disease, cognition, cognitive assessment

## Abstract

Cognitive and behavioral abnormalities are recognized as an integral part of Motor Neurone Disease (MND) and occur at all stages of the disease. The early detection of cognitive and behavioral symptoms in MND is critical. Such symptoms are only reported when we explicitly ask, evaluate, document, and assess. In the National Institute for Health and Care Excellence (NICE) MND guideline (2016), formal cognitive and behavioral assessment is incorporated in MND management and is fundamental to providing appropriate care to pwMND. Cognition is explicitly stated in 14 separate recommendations in the guidelines. The NICE guidelines therefore constitute pre-defined standards which we audited. This audit highlights that health professionals increasingly recognize the significance of cognitive screening in MND and follow more structured approaches in implementing this compared to previous years.

## Background

Cognitive and behavioral abnormalities are recognized as part of Motor Neurone Disease (MND). Approximately 15% of people with MND (pwMND) fulfill diagnostic criteria for fronto-temporal dementia (typically behavioral variant), while milder changes in behavior and cognition affect a further 35% of pwMND (1,2). Cognitive and behavioral impairments have important functional implications for pwMND and their families: they increase caregiver burden, reduce survival, and impact care planning (3). These impairments may not be readily apparent at clinical interview, hence the need to assess using standardized measures. In the National Institute for Health and Care Excellence (NICE) MND guideline (2016), cognitive and behavioral assessment is incorporated in MND management and is fundamental to providing appropriate care to pwMND (Table 1) (4,5,6).

**Table 1:** Audit standards.

**Standard 1.** Following diagnosis of MND, cognitive screening is recommended in all patients.
**The NICE recommendations are (4):**
At diagnosis, and if there is concern about cognition and behavior, explore any cognitive or behavioral changes with the person and their family members and/or carers as appropriate. If needed, refer the person for a formal assessment in line with the NICE guideline on dementia. [new 2016]
The multidisciplinary team (MDT) should assess, manage and review the following areas, including the person's response to treatment: … cognition and behaviour
Tailor all discussions to the person’s needs, considering their communication ability, cognitive status and mental capacity
**Standard 2**. Care planning should be adapted for people with cognitive impairment and behavior change.
**The NICE recommendations are:**
Planning of end of life care
Be sensitive about the timing of discussions and consider the person's current communication ability, cognitive status and mental capacity
Think about discussing advance care planning with people at an earlier opportunity if you expect their communication ability, cognitive status, or mental capacity to get worse. Cognitive impairment and the number of reported behavioral features are significantly related to advancing disease stage and are more likely to occur to those with cognitive changes at onset (5,6).
2.Use of gastrostomy
Before a decision is made on the use of gastrostomy for a person with MND who has frontotemporal dementia, the neurologist from the MDT should assess the following:
The person's ability to make decisions and to give consent
The severity of frontotemporal dementia and cognitive problems
Whether the person is likely to accept and cope with treatment
3.Non-invasive ventilation
Before a decision is made on the use of non-invasive ventilation for a person with a diagnosis of frontotemporal dementia, the MDT together with the respiratory ventilation service should carry out an assessment that includes: the person's capacity to make decisions and to give consent, the severity of dementia and cognitive problems

(5) Crockford et al. Neurology 2018; (6) Elamin et al., Neurology 2013.

The Edinburgh Cognitive and behavioral ALS screen (ECAS) has been specifically developed and validated (7,8). In Scotland, assessment of pwMND is provided either via neuropsychology or members of the multidisciplinary team (MDT), usually the clinical nurse specialist. Furthermore, Scotland benefits from an integrated national healthcare team for MND closely aligned to the Scottish MND register (relaunched in 2015 as Clinical Audit Research and Evaluation of MND – CARE-MND) (9). This is an electronic platform for prospective, population-based research.

We conducted an audit to analyze “real-world” experience of cognitive assessment in pwMND in Scotland. Using the NICE guidelines, we measured whether we follow best practice.

## Objectives

To audit cognitive assessment in pwMND in Scotland against two predetermined standards by NICE:

- Following MND diagnosis, cognitive screening is recommended in pwMND.
- Care planning should be adapted for people with cognitive impairment and behavior change.

## Methods

During 2016, several educational activities occurred within Scotland and the UK to encourage cognitive and behavioral screening of MND as part of routine assessment. At the end of 2016, we presented the preliminary results throughout Scotland and identified obstacles to screening.

Data were captured from the CARE-MND Register. Two time periods were audited:

1. January 2015 – December 2016
2. January 2017 – December 2017.

## Results

(1) Between January 2015 and December 2016, 393 new cases were captured (Table 2). ECAS was undertaken in 36% (*n* = 140). Almost 33% (*n* = 131) did not have any form of cognitive assessment. No data were recorded in the remaining cases (31%; *n* = 122); this is likely attributed to the following: there is no funded clinical neuropsychology in some areas; long-term staff sickness; in some regions, cognition was considered satisfactory and therefore cognitive screening was not offered.

**Table 2:** Patient demographics.

	Audit phase 1	Audit phase 2
Median age of onset	67 (Median Abs deviation = 8)	66 (Median Abs deviation = 8.5)
% males, % females	63%, 37%	61%, 39%
Median disease duration (in months)	27 (median Abs deviation = 13)	25 (Median Abs deviation = 13)

**Figure 1:**
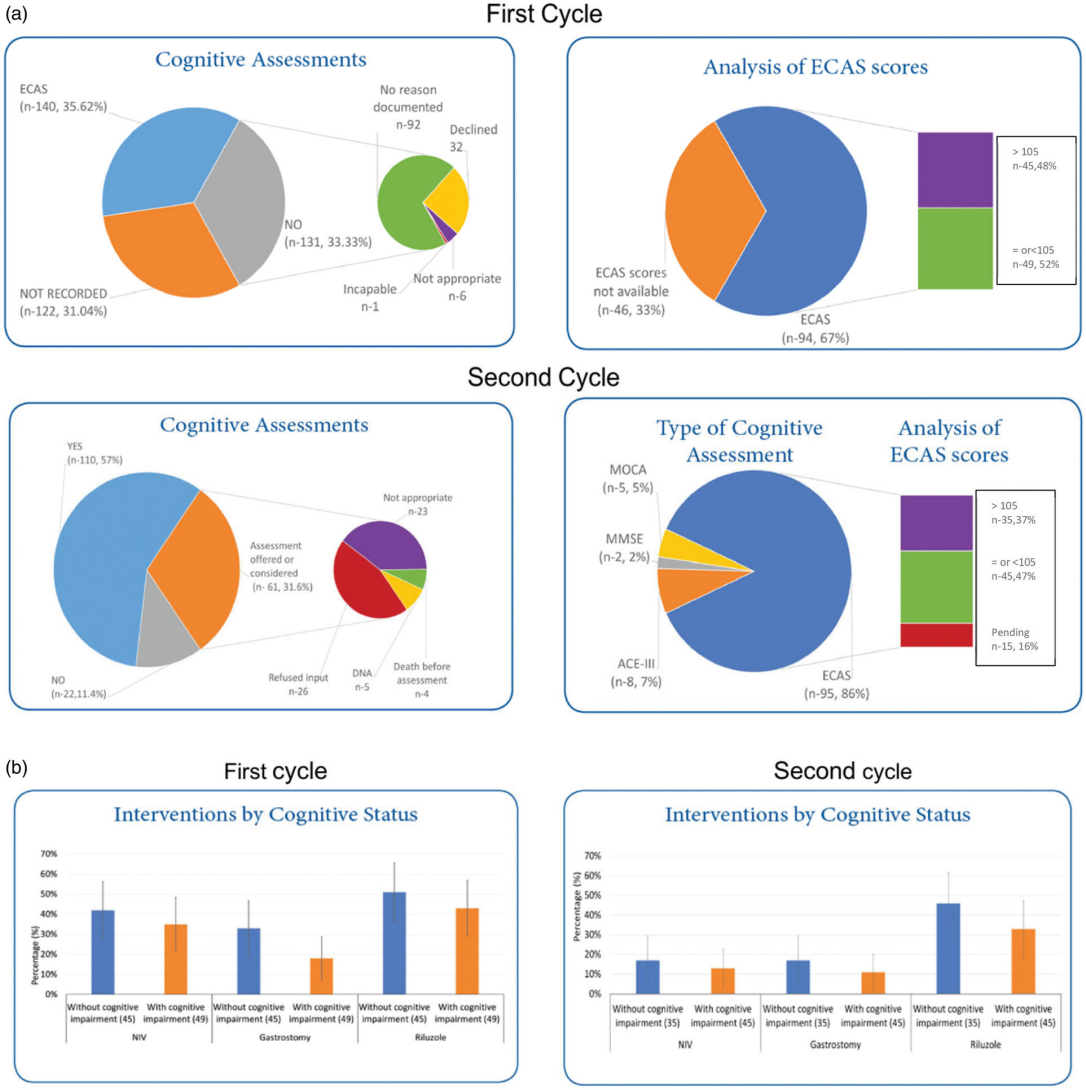
(a) Implementation of cognitive assessments Phase 1 & 2. In the UK, the cutoff for the ECAS total is 105. A score AT or BELOW 105 suggests that a person may have cognitive impairment. “Pending” were the cases where ECAS was undertaken but the data were not yet interpreted or inputted to the CARE-MND platform. (b) Interventions by cognitive status.

Total ECAS scores were available in 67% (*n* = 94) and 50% had cognitive impairment (defined by the cutoff total ECAS scores) (Figure 1(a)). There was a non-significant trend toward fewer interventions (non-invasive ventilation, gastrostomy, riluzole) in cognitively impaired patients (Figure 1(b)).

(2) In 2017, data were available from 193 incident cases (Table 2). Around 57% (*n* = 110) underwent cognitive screening, with ECAS performed in 86% (*n* = 95), and other forms of cognitive assessments in the rest. Total ECAS scores were available in 85% (*n* = 80); 54% of those had cognitive impairment (Figure 1(a)). Comparing those with and without cognitive impairment on treatment, there was no statistically significant difference (Figure 1(b)).

## Discussion

This audit highlights that health professionals increasingly recognize the significance of cognitive screening in MND and follow more structured approaches compared to previous years. Cognitive testing increased by 21% and ECAS was the most commonly performed assessment. Of those assessed, 56% were cognitively impaired, consistent with previous population studies.

During the second cycle, cognitive assessments were not recorded for 11.4%. The reasons include: (1) delay in data input in the CARE-MND platform, (2) ECAS was not collected because it was too soon after diagnosis. We offer pwMND follow-up appointments with an MDT member 4–6 weeks post-diagnosis. This delay prevents misconstruing emotional overburdening with cognitive impairment.

Regarding treatment, there was a non-significant trend toward fewer interventions in cognitively impaired patients and other groups have also identified this (10). Those with cognitive change or FTD may have difficulty coping with NIV (11). Larger cohorts are required confirming the association between interventions and cognitive impairment in MND. Specifically, more data are needed on NIV compliance, the role of patients, carers and physicians in the decision-making process, and end-of-life practices in pwMND with cognitive change. Our future work involves investigating the profile of supportive/palliative interventions in pwMND with mild or moderate cognitive impairment, behavioral changes and the frontotemporal syndrome. Further studies should elucidate how to ensure appropriate interventions are not inadvertently denied.

Additional pathways should be developed for cognitive/behavioral screening for pwMND such as: (1) Masterclasses/training days to enhance health care professionals’ knowledge in ECAS; (2) Establishment of dedicated ECAS clinics or incorporating psychologists into clinic; (3) Ongoing support and access to neuropsychology services. Neuropsychological intervention helps the MDT manage the particularly complex cases.

Finally, the online register has contributed to accurate and effective data capturing. CARE-MND Platform is a national resource with an average case ascertainment coverage of 99% (7). Centralizing data from across Scotland are unique and beneficial for patient care. Ongoing multi-disciplinary collaboration is essential for mastering data capture, attaining compliance with evidence-based recommendations and continuing quality improvement.

## Data Availability

Requested for access to data should be addressed to the corresponding author and the CARE-MND Consortium.
